# Clinical outcome of endovascular coil embolization for cerebral aneurysms in Asian population in relation to risk factors: a 3-year retrospective analysis

**DOI:** 10.1186/s12893-020-00756-1

**Published:** 2020-05-14

**Authors:** Saima Ahmad

**Affiliations:** Pakistan Institute of Neurosciences, Neurointerventional Department, Lahore General Hospital, Ferozpur Road, Lahore, Pakistan

**Keywords:** Commodities, Smoking, Hypertension, Endovascular coiling

## Abstract

**Background:**

Long term results of endovascular coiling are yet scarce. This study reviews the impact of comorbidities on the success of endovascular coiling of both ruptured and unruptured intracranial aneurysms. Endovascular treatment has become thetreatment of choice after the ISAT trials. Independent risk factors that influence recovery are age, sex, smoking, and hypertension.

**Methods:**

This is a 3-year retrospective analysis, performed to assess the clinical and radiological outcome of patients with cerebral aneurysms treated with detachable coils in an Asian population with relation to comorbidities including smoking and hypertension with age and gender as mediators. From July 2015 to August 2018, a total of 297 consecutive patients (mean age: 45.5 years) with cerebral aneurysms both ruptured and unruptured who were treated at a single center with endovascular coiling procedures are included in the study. Clinical information and radiological outcomes were evaluated on regular follow-ups and telephonic interviews. A modified Rankin Scale was used to measure the clinical outcomes in patients.

**Results:**

We have found that smoking harmed clinical outcome, with smokers 35% less likely to recover, while hypertension played a smaller role with only 15%. It was found that while aneurysms are more prevalent in women than men, women not only have a higher chance of getting an aneurysm but also have poorer outcomes. Similarly, patients who were above the age of 40 had a lower chance of recovery compared to younger patients due to comorbidities irrespective of gender.

**Conclusion:**

Asian sub-continent has different genetic markers that lead to poorer outcomes of aneurysms in women, while outcomes are similar in men and women in developed nations. Smoking does not play a major role in women’s recovery. Men with comorbidity seemed to be at higher risk and age played a major role in their recovery.

## Key points

1. The study examines the long-term clinical and radiological outcome of endovascular coil embolization for cerebral aneurysms about age, gender, and comorbidities.
2. Men in their 20s and 30s have the best recovery, with comorbidities playing a negligible role.
3. Asian sub-continent has a different genetic marker that lead to poorer outcomes of aneurysms in women, while outcomes are similar in men and women in developed nations. This can also perhaps be due to cultural factors.

## Background

Subarachnoid hemorrhage secondary to ruptured intracranial aneurysms is a life-threatening condition with an incidence rate of 6–7 per 100,000 people per year in most ethnic populations [1]. Since the inception of endovascular embolization in 1991, direct coiling has become an established technique for the treatment of patients with both ruptured and unruptured intracranial aneurysms [2, 3]. In 2002, results of the International Subarachnoid Aneurysm Trial [1] demonstrated that there was a better clinical outcome of Endo Vascular Therapy (EVT) over surgical clipping of ruptured intracranial aneurysms [4].

Our institution performs coiling on 90% of aneurysm patients. Only a few studies have reported the short and midterm results of endovascular coiling, but long-term studies remain elusive. In approximately 20% of patients, the coiled aneurysms reopen in follow up [5]. The angiographic results of coil occlusion can be classified into complete, near-complete or incomplete using a modified Raymond classification scale. Occlusion is “near-complete” or “subtotal” when the sac is occluded but a neck remnant remains. “Incomplete occlusion” can be defined as an aneurysm having loose packing and partial opacification of the aneurysm sac. The distinction between small recurrences (a small change in packing) and full recanalization is determined by the performing physician. Recanalization, as a consequence of coil compaction due to high arterial blood flow or aneurysm growth, was considered to require a second treatment because of the high risk of rupture [6].

Smoking is known to increase the risk of Sub Arachnoid Hemorrhage (SAH) and brain infarction when baseline characteristics of case and control patients were kept constant, smoking status directly and significantly correlated with the onset of the disease and established smoking as an independent risk factor in SAH [4]. Smoking greatly impacts the need for patient care during endovascular treatment and negatively impacts long term recovery, however, long-term recovery impact has not been measured [7].

The last comorbidity measured in this study is hypertension. Patients presenting with SAH who had high grades of Hunt & Hess Grade (HHG) and Intra Ventricular Hemorrhage (IVH) have been associated with poor outcomes with statistical significance. This indicates the need for careful consideration when opting for aggressive therapy. However, when looking at elderly patients with Unruptured Intracranial Aneurysm’s (UIA) the results of treatment were found to be consistent regardless of treatment modality and aggressive treatment can still be considered in UIA cases. It has been found that high-grade HHG, increased age, and intra-ventricular hematoma have poor clinical outcomes as compared to patients without these factors. In such cases, it is recommended to place external ventricular drainage before aneurysm securing intervention to improve short-term outcomes [8].

Other studies found that patient characteristics such as higher age, the female gender has poor outcomes of SAH. Primary risk factors are old age, female gender and smoking while hypertension plays a higher role in younger patients but does not have much impact on elderly patients. There is no evidence of rupture risk and older age and descent has not been found in the literature. This leaves a gap in the available literature that can be filled by looking at the population of the Asian sub- continent over an extended period and clubbing these various risk factors into a singular multivariate analysis. The aim of this study is to find the correlation between age, gender, hypertension and smoking with clinical outcome of patients who underwent endovascular treatment for aneurysms.

## Methods

### Patients data

This study was approved by the hospital’s ethical review board and the institutional review board. Consent forms for all patients were obtained and in the case of a minor, a guardian was asked to sign on their behalf. All patients with ruptured and unruptured intracranial aneurysms initially treated with endovascular coiling with or without remodeling technique between July 2015 to August 2018 are included in this study. The information was obtained from a neuro-interventional procedure logbook maintained at the institute. The period chosen allows an opportunity for retrospection of at least 3 years of clinical and angiographic follow-ups. The patient whose aneurysms had prior clippings done were excluded from the study. If multiple aneurysms were present, the ruptured aneurysm was determined from the distribution of subarachnoid hemorrhage and intraventricular blood. If the site of the hemorrhage could not identify the ruptured aneurysm, we treated all aneurysms in a single session. Patients’ age, sex, the presence or absence of smoking and hypertension, number of aneurysms, whether or not it ruptured, the location of the aneurysm and any recurrences were recorded. Additionally, two-thirds of the patients with hypertension were diagnosed at the time of ictus. The recovery of patients was gauged clinically with the Modified Rankin scale and radiological outcome was measured through Raymond classification and both were also During the period of this study, the overall management strategy was endovascular coiling as the first treatment option, if feasible. Each case was discussed and consensus reached by members of the neuro-interventional team. Patients with large intracerebral hemorrhage associated with a ruptured aneurysm generally underwent emergency evacuation before coiling. Ruptured intracranial aneurysms were generally treated within 24 to 72 h of rupture. In all endovascular treated aneurysms, detachable coils were used to occlude as much as of aneurysm as possible in a safe manner. In the case of aneurysms with wide necks or unfavorable shapes, we used the assisted technique of double catheter, balloon or stent-assisted coiling. After the completion of coil embolization, we divided the angiographic findings into three classes using the Modified Raymond Scale: complete occlusion, neck remnant, and residual aneurysm.

### Clinical parameters and long term follow up

Baseline clinical information was retrospectively abstracted from hospital charts and stratified. Computed tomography scans and digital subtraction angiograms were analyzed before deploying the first coil. Aneurysm sac diameter, neck width, and dome to neck ratio was analyzed before prior coiling. The use of remodeling techniques (balloon or stent) was also decided on angiography. Clinical outcome was defined as a deterioration of > 0 on the mRS and any deaths related to the treatment. Clinical outcome and neurological status were evaluated in detail during every outpatient visit as well as via follow-up overcall.

### Angiographic analysis and long term follow up

Angiographic follow up is not routinely done for clipped aneurysms, however, coiled aneurysms need follow-up, but the recommended length of follow up has not been established. Age of the patient, comorbidities, size and shape of the aneurysm, location, rupture status and occlusion grade were taken into account when deciding the follow up duration by Sprengers et.al (2008) [3] who suggested that adequately occluded aneurysms at 6 months after coiling do not need imaging to follow up, except aneurysms that are partially thrombosed or larger than 15 mm. The standard operating procedure at our institution is having first follow up magnetic resonant angiography (MRA) after 6 months, followed by another at 1 year and then serially for 3 years. In patients with incomplete initial aneurysm occlusion or reopening of the aneurysm over time, different intervals for angiographic follow up were chosen because additional treatment was considered in those patients. In other cases, if post coiling 6 months MRA showed some residual or recurrent neck, we have a fixed protocol that entails repeating the angiogram to confirm the findings of MRA. Similarly, Soize et al. (2016) has also showed that imaging follow-up of intracranial aneurysms treated via endovascular methods is imperative towards the goal of detecting regions at risk of bleeding. Even though it is very important in the first year, follow-ups are beneficial. Since no exact guidelines exist, the frequency of monitoring and the imaging modality to use is adapted on a case by case basis (Soize et al., 2016). As is the case with Soize et al. (2016), this study also relied on MRA as a suitable imaging modality after coil emboliation.

### Outcome measures

The outcome was assessed by an independent neurosurgeon and neuro-interventionalist by using the modified Rankin Scale. The primary outcome measure was functional outcome at discharge and at first, follow up after 15 days and then every 6 months until the end of the 3-year period.

### The methodology of analysis

IBM SPSS Version 25 was used for the analysis of data-set collected. A specialist statistician’s services were acquired for the tabulation of data, the decision of analysis to be conducted and for the interpretation and analysis of data findings.

Multi-variate auto-regressions were run keeping all factors in a single equation. Furthermore, smoking, hypertension, age group, and sex were all individually tested against clinical outcomes. As well as conducting a chi-square test to see the results of various permutations such as age and hypertension, age and smoking, sex and age, etc. Cronbach-Alpha, p, and p2 values were also determined to ratify the statistical significance of results. A linear regression equation was made and run to find the impact of factors on clinical outcomes and the equations can be seen as follows:

$$ \mathrm{Clinical}\ \mathrm{Outcome}=\mathrm{Age}+\mathrm{Sex}+\mathrm{Hypertension}+\mathrm{Smoking}+\mathrm{Residual} $$

Clinical outcomes were on the Modified Rankin Scale with a rating from 0 to 6, 0 being perfect recovery and 6 being death. Age was divided into intervals of 10 years as follows < 20 21–30 30–40 40–50 50<. Finally, sex, hypertension, and smoking were kept as binary variables with sex being male or female and hypertension and smoking being present or absent.

### Statistical analysis

We first tabulated all the data into both Excel and SPSS. The excel files were converted to pivot tables (Tables 1, 2 and 3), which we used to draw several key conclusions as well as generate all the graphical outputs and figures for this paper. We saw the correlations between age and hypertension, smoking and age, age and gender, hypertension and gender and finally smoking and gender. We also made a regression equation and ran a regression in SPSS to test our data for both validities of the model and secondly to be able to understand what impact the factors we were studying namely; age, gender, hypertension, and smoking had on the clinical outcome of patients. The R2 value of the regression was 0.69 which means that the model was quite highly accurate given that this study utilized real-world data from the only possible authority on the subject in the country. The ANOVA residual value factor was found to be quite high nearly 3 times as high as the regression values which leads us to believe that greater exploration into the subject matter is required and there are other factors that were not considered in this study and must be done so in order to find a more accurate interpretation of what factors contribute to patient recovery. Factors not considered are discussed in the next portion of this paper and can be the basis of future research on the subject.

**Table 1:**
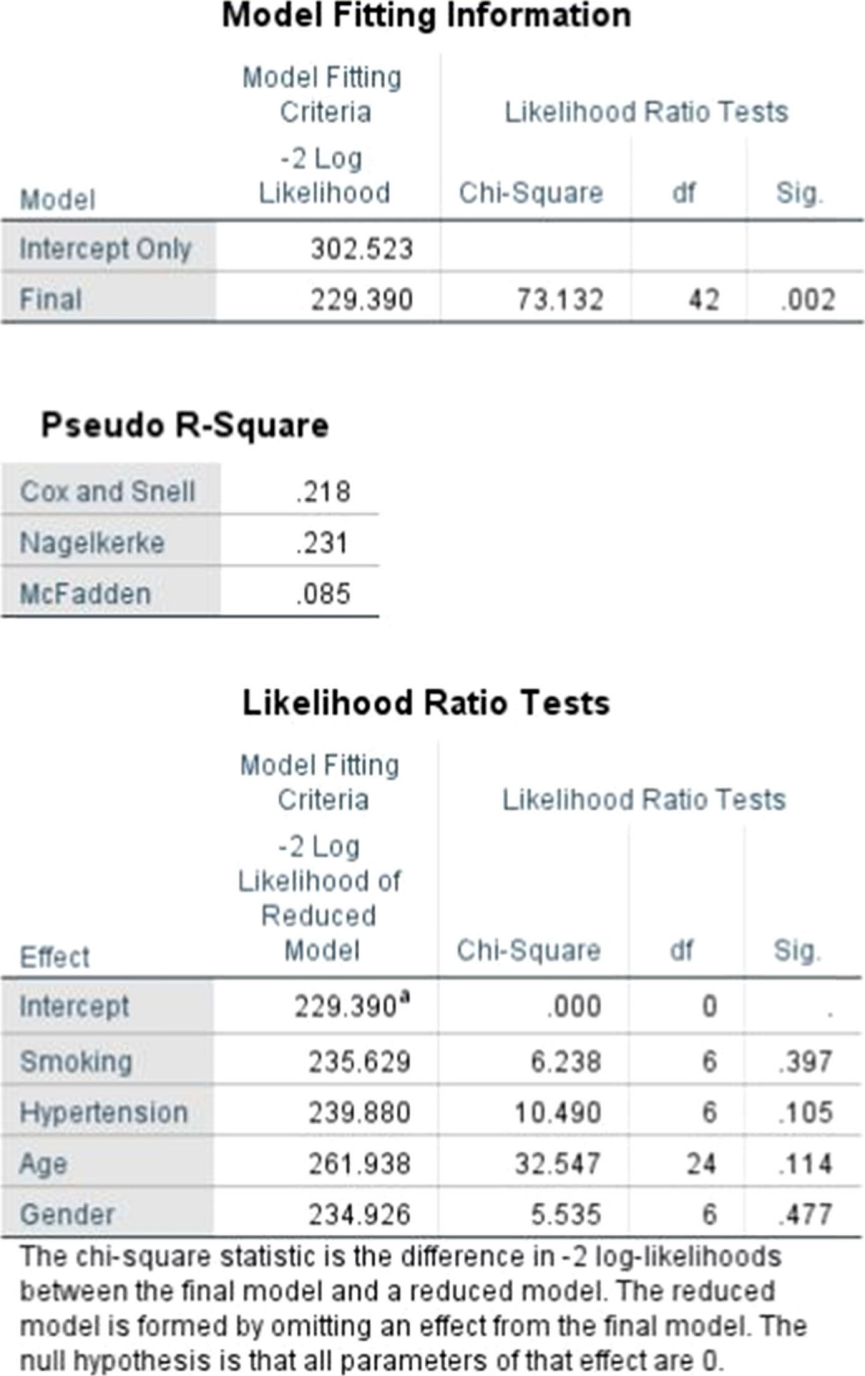
Model fitting and likelihood ratio tests of data, showing that the model fitting and likelihood models are significant at a *p* < 0.05 (*p* = 0.002)

**Table 2:**
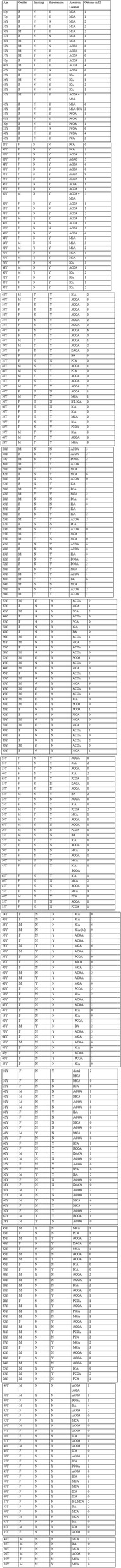
Patient demographics and key variables

**Table 3:**
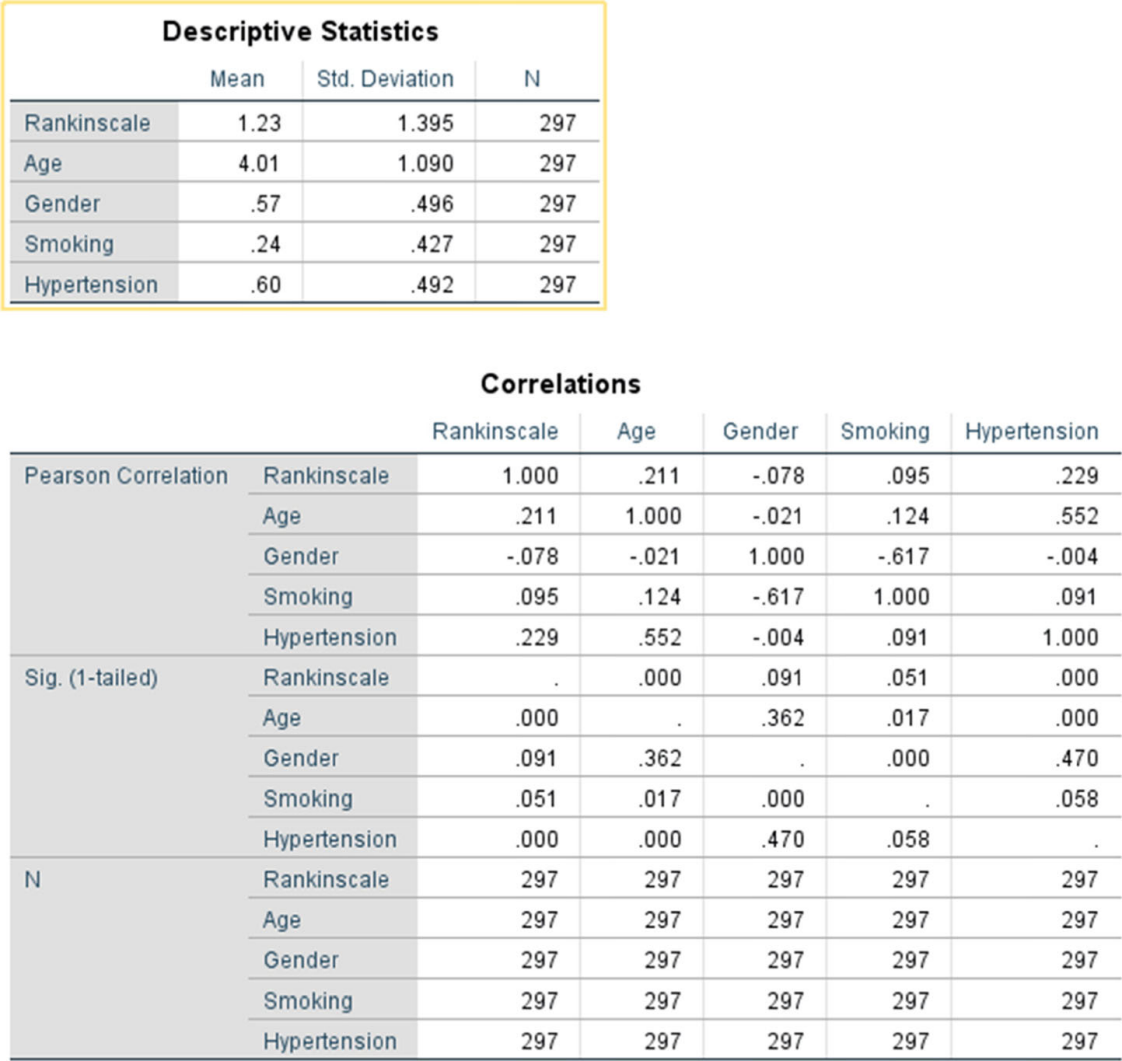
Descriptive statistics and correlational analysis of data (showing the Pearson’s correlation between the shown variables and their respective *p*-values and the number of entries in the sample)

## Results

### Descriptive statistics

The study had a near equal mix of males and females, with female patients being a majority at 57.6% of the total. The average age of patients in the study was 42 with the majority of the patients being within the age range of of 40–50 years old (38.0%). The standard deviation in the age group was 1.09 meaning approximately 10 years. The average patient had a modified Rankin scale result of 1.2 with a standard deviation of 1.4, meaning that the overall outcome of patients who had coiling done was positive. Smoker to non- smoker ratio in the study was 24:76 meaning that for every 100 patients 24 were smokers. Hypertension, on the other hand, was highly prevalent with a ratio of 40:60 with every 60 out of 100 recorded patients having a history of hypertension. Additionally, the proportion of hypertensive patients by gender was nearly the same, with about 59.6% of the females and about 60.3% of the males having hypertension. However, a gender difference was seen in the proportion of smokers, none of the females reporting to be smokers and about 57.1% of males being smokers.

### Statistical analysis results

When looking at the factors considered the value age group had a factor of 0.150 meaning that higher a patients age the less likely he was to fully recover from the aneurysm as age was broken into groups and higher age groups assigned a higher numerical value. Smoking played a much more critical role with a factor of 0.103 meaning that non-smokers had a better recovery than smokers as non-smokers were assigned the numerical value of 0 while smokers had a value of 1.

**Fig. 1:**
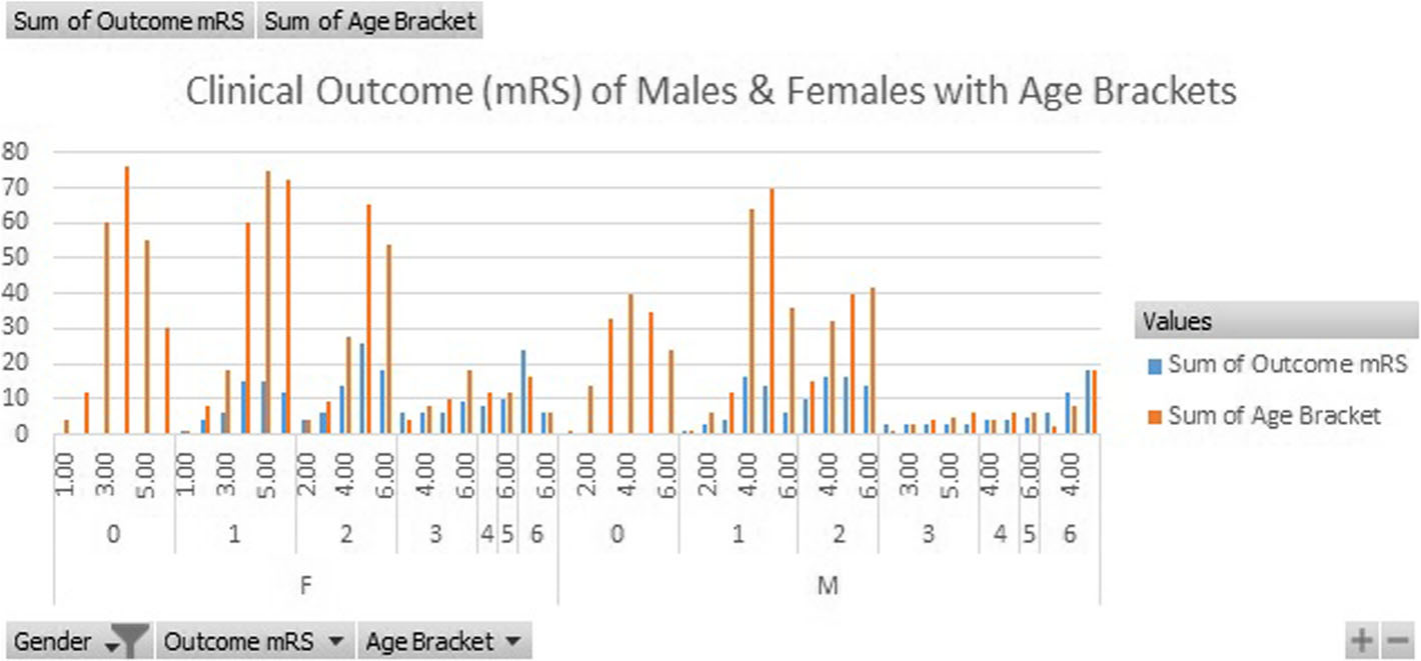
Graphical representation of clinical outcomes in both genders sorted by age groups

**Fig. 2:**
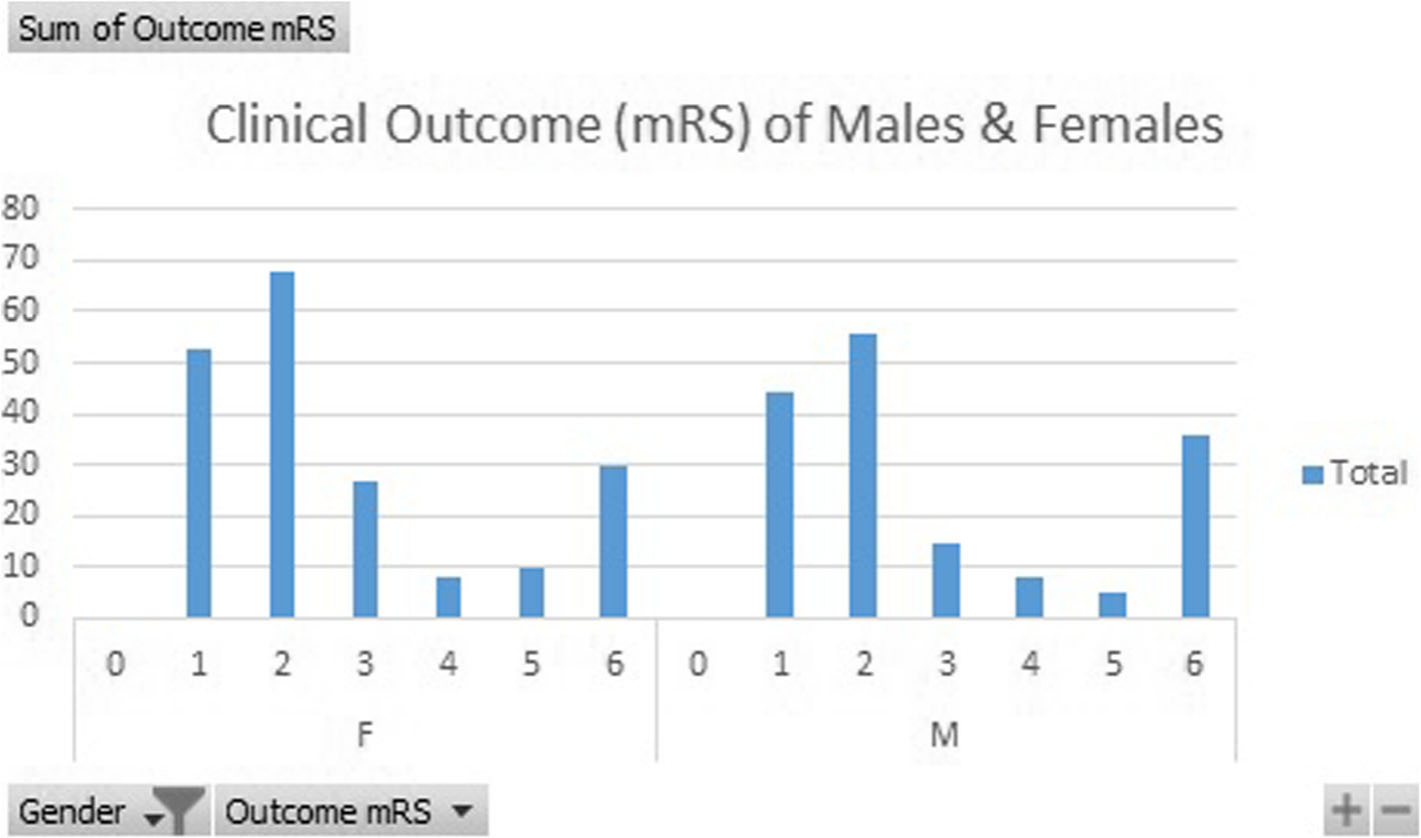
Bar graph representing clinical outcome in both genders

Hypertension presence had a far more significant impact on recovery with a factor value of 0.457 meaning that those with hypertension were more than 45% more likely to have poorer outcomes (Tables 1 and 2). Lastly, the only factor considered in the study to have an inverse negative impact was gender having a factor value of − 0.155 meaning that women simply due to their gender had poorer odds of recovery as compared to men. Finally, when age and gender were fixed against the Rankin Scale and we used hypertension and smoking as covariates we were able to find very interesting patterns. For patients who had optimal recovery smoking played a negative factor of 0.77, hypertension a factor of − 1.82 and the most impacted age group was that of patients in the age between 40 and 50 with a factor of − 0.815 (Table 3). Gender was not seen to play a very impactful role when there was complete recovery, however, when we reviewed patients who had a Rankin scale rating of 5, gender played a major role with a factor of 1.07 and age group not being very impactful with patients in the age between 30 and 50 is similarly impacted. Hypertension and smoking both played equally impactful roles with factors of 12 and 17 respectively. Additionally, it was seen that clinical outcomes varied by age groups (Fig. 1) and by gender (Fig. 2).

## Discussion

This study discusses the importance of endovascular coiling as an interventional method in preventing death and dependency in both ruptured and unruptrued aneurysms. Molyneux et al. (2014) concluded the same in their research, which analyzed data from about 1644 patients, where it was found that the probability of death and dependency was significantly greater in the neurological group as compared to the endovascular group. Additionally, although rebleeding was more likely after endovascular coiling than the neurological clipping, this risk was small and the chance of disability free survival was higher (Molyneux et al., 2014). Additionally, Gallas et al. (2009) also showed that only 7% of aneurysms from 1036 patients needed to be retreated and that the coiling method gives a good long-term level of occlusion (Gallas et al., 2009). Given this information, this paper looks into the uses of endovascular coiling since this method has higher chances of survival amongst the patients. Furthermore, in a study conducted on the Australian population, it was found that there is a long-term efficacy of endovascular coiling, particularly for posterior circulation aneurysms (Wilson, Drackford and Holt, 2015).

It has long been established that smoking is bad for health. However, it has been found that smoking is a factor that contributes to the rupture of aneurysms. A study carried out on an Asian population concluded that hypertension and smoking may be causal risk factors which might modify the effect of already present genetic factors that could increase the likelihood of aneurysms (Koshy et al., 2019).

Aneurysms are usually preceded by migraine and excessive smoking. Hypertension does not, however, impact the probability of a rupture [9]. The size of the aneurysm has also been seen in literature to contribute to aneurysm rupture and smokers who have necks larger than 4 mm are 20% more likely to have a thromboembolic event during endovascular coiling procedure.

Similarly, age also plays a role in thromboembolic events with patients above the age of 50 having a higher likelihood of rupture [10]. Similarly, age groups of patients when younger than 30 or older than 50 have an equal likelihood of poor recovery but patients between the ages of 30–50 have the best recovery rates. Our study was not focused on the rupturing of aneurysms but the recovery and quality of recovery of patients after having an embolic event. We found that endovascular treatment of aneurysms has highly successful results and gender plays the most pivotal role in such cases.

Hypertension in elderly patients plays a huge role in their recovery from ruptured aneurysms, it was found by our study that hypertension and old age had a greatly negative impact on recovery after coiling and this is in concurrence with the findings of various available literature. That has shown that elderly patients with comorbidity of hypertension have statistically significant poorer outcomes and thus need to be treated more aggressively with greater post-procedural care and regular follow-ups [8].

Past studies showed that Europeans women over the age of 60 have excellent to good outcomes however our study showed the inverse to be true [10]. Asian women are seen to have the poorest recovery and high morbidity rate when compared to various age groups and comorbidities. Another study found that men were independently associated with larger aneurysms and that height and size ratio played a great role in outcome and sex was not a risk factor for patients. Our study did not take into account the size and height of aneurysm and perhaps this could be a factor that contributed to the larger residual value. While the study by Lin, Chen et.al showed that ruptured aneurysm was more common in men our study found that it was women who had higher rates of aneurysms [10]. Similarly, the study showed that men and women showed similar recoveries when age was held constant again something that our results are in stark contradiction of. This leads us to believe that Asian populations especially the Asian sub-continent have a different genetic marker that leads to the greater prevalence of aneurysms in women, there are a generally lower life expectancy and weaker life strength of women in the Asian sub-continent and it is perhaps cultural factors that are the reason for poorer outcomes in women. Another study looking into the genetic risk factors pertaining to anuerysms concluded that certain ethnicities are more prone to such aneruems as compared to others (Alg et al., 2013). This might certainly be true for Asian populations as well. However, there is little to no research on the genetic risk factors associated with aneurysms in the Asian population. Further research is needed to elucidate the effects of the presence, or absence, of certain genes in causing aneurysms in the Asian population.

## Conclusion

This study is the first of its kind ever conducted in Asia at the only center in the country that is dedicated to NeuroIntervention and has the facility for coiling procedures. Very few studies, in general, have been conducted on the Asian population in regard to aneurysms. We found that women in the sub-continent have not only far higher frequency of aneurysms but also poorer outcomes. Poor recovery is compounded by the presence of comorbidity of hypertension. Smoking does not play a major role in women’s recovery or at least this fact was not discernable by this study most likely due to a lack of data as most women in Asia due to religious and cultural reasons are mostly non-smokers. Men with comorbidity of smoking and hypertension were seen to be at higher risk and age played a major role in recovery. Men in their 20s and 30s saw the best recovery rates which are highly encouraging with hypertension and comorbidity only having a minor impact on their recovery. It was seen however that men who were 40+ in age had far more negative impacts of smoking and hypertension in their recovery and showed very poor results.

## Data Availability

All data was gathered at a single institution. The data sets used or analyzed during the current study are available from the corresponding author on reasonable request for review by the peer reviewers.

## Abbreviations

EVT: Endo Vascular Therapy
HHG: Hunt & Hess Grade
IVH: Intra Ventricular Hemorrhage
MRA: Magnetic Resonant Angiography
SAH: Sub Arachnoid Hemorrhage
UIA: Unruptured Intracranial Aneurysm

## References

[1] Darsaut T, Jack A, Kerr R, Raymond J. International subarachnoid aneurysm trial–ISAT part II: study protocol for a randomized controlled trial. Trials. 2013;14(1):156. 10.1186/1745-6215-14-156. 10.1186/1745-6215-14-156 PMC3680206 23714335

[2] Pyysalo L (2012). Long-term outcome of patients with Embolized intracranial aneurysms.

[3] Van Rooij WJ, Sluzewski M (2009). Opinion: imaging follow-up after coiling of intracranial aneurysms. Am J Neuroradiol.

[4] Xu B, Ji Q, Zhang Y, Shen L, Cao M, Cai K (2017). Postoperative blood pressure variability exerts an influence on clinical outcome after coil embolization of ruptured intracranial aneurysms. Neurol Res.

[5] Pyysalo LM, Keski-Nisula LH, Niskakangas TT, Kähärä VJ, Öhman JE (2010). Long-term follow-up study of endovascularly treated intracranial aneurysms. Interv Neuroradiol.

[6] Gallas S, Januel AC, Pasco A, Drouineau J, Gabrillargues J, Gaston A (2009). Long-term follow-up of 1036 cerebral aneurysms treated by bare coils: a multicentric cohort treated between 1998 and 2003. Am J Neuroradiol.

[7] Pierot L, Cognard C, Anxionnat R, Ricolfi F, Investigators C (2010). Ruptured intracranial aneurysms: factors affecting the rate and outcome of endovascular treatment complications in a series of 782 patients (CLARITY study). Radiology.

[8] Park JH, Kim YI, Lim YC (2014). Clinical outcomes of treatment for intracranial aneurysm in elderly patients. J Cerebrovasc Endovasc Neurosurg.

[9] Williams LN, Brown RD (2013). Management of unruptured intracranial aneurysms. Neurology: Clin Pract.

[10] Park J, Kim Y, Lim Y. Clinical outcomes of treatment for intracranial aneurysm in elderly patients. J Cerebrovasc Endovasc Neurosurg. 2014;16(3):193. 10.7461/jcen.2014.16.3.193. 10.7461/jcen.2014.16.3.193 PMC4205244 25340020

